# Characterization of three *lamp* genes from largemouth bass (*Micropterus salmoides*): molecular cloning, expression patterns, and their transcriptional levels in response to fast and refeeding strategy

**DOI:** 10.3389/fphys.2024.1386413

**Published:** 2024-04-05

**Authors:** Yan-Lin Yang, Wan-Hong Zeng, Yong Peng, Shi-Yu Zuo, Yuan-Qi Fu, Yi-Ming Xiao, Wen-Li Huang, Zheng-Yong Wen, Wei Hu, Yu-Ying Yang, Xiao-Feng Huang

**Affiliations:** ^1^ Hubei Key Laboratory of Waterlogging Disaster and Agricultural Use of Wetland, Yangtze University, Jingzhou, China; ^2^ School of Animal Science, Yangtze University, Jingzhou, China; ^3^ Key Laboratory of Sichuan Province for Fishes Conservation and Utilization in the Upper Reaches of the Yangtze River, Neijiang Normal University, Neijiang, China

**Keywords:** largemouth bass, lysosomes-associated membrane proteins, tissue expression, fasting, re-feeding

## Abstract

Lysosomes-associated membrane proteins (*LAMPs*), a family of glycosylated proteins and major constituents of the lysosomal membranes, play a dominant role in various cellular processes, including phagocytosis, autophagy and immunity in mammals. However, their roles in aquatic species remain poorly known. In the present study, three *lamp* genes were cloned and characterized from *Micropterus salmoides*. Subsequently, their transcriptional levels in response to different nutritional status were investigated. The full-length coding sequences of *lamp1*, *lamp2* and *lamp3* were 1251bp, 1224bp and 771bp, encoding 416, 407 and 256 amino acids, respectively. Multiple sequence alignment showed that LAMP1-3 were highly conserved among the different fish species, respectively. 3-D structure prediction, genomic survey, and phylogenetic analysis were further confirmed that these genes are widely existed in vertebrates. The mRNA expression of the three genes was ubiquitously expressed in all selected tissues, including liver, brain, gill, heart, muscle, spleen, kidney, stomach, adipose and intestine, *lamp1* shows highly transcript levels in brain and muscle, *lamp2* displays highly expression level in heart, muscle and spleen, but *lamp3* shows highly transcript level in spleen, liver and kidney. To analyze the function of the three genes under starvation stress in largemouth bass, three experimental treatment groups (fasted group and refeeding group, control group) were established in the current study. The results indicated that the expression of *lamp1* was significant induced after starvation, and then returned to normal levels after refeeding in the liver. The expression of *lamp2* and *lamp3* exhibited the same trend in the liver. In addition, in the spleen and the kidney, the transcript level of *lamp1* and *lamp2* was remarkably increased in the fasted treatment group and slightly decreased in the refed treatment group, respectively. Collectively, our findings suggest that three *lamp* genes may have differential function in the immune and energetic organism in largemouth bass, which is helpful in understanding roles of *lamps* in aquatic species.

## 1 Introduction

Lysosomes, which may originate from pre-existing endolysosomes or autolysosomes (Mahapatra et al., 2021), are spherical bodies with amorphous of 50–500 nm diameter, single membrane, terminal degradation vesicular structures organelles, and are found in almost all eukaryotic cells (Debjyoti et al., 2014; Rondón-Barragán et al., 2017). Lysosomes are responsible for the degradation of cellular macromolecules (such as carbohydrates, proteins, lipids, and nucleic acids) into their constituent building blocks, which are generally obtained via phagocytosis or endocytosis, or autophagy (Alessandrini et al., 2017; Baba et al., 2020). Accumulating evidence indicated that lysosomes act as a metabolic signalling centre and play an essential role in numerous physiological progresses, such as, nutrient sensing (Perera and Zoncu, 2016; Lawrence and Zoncu, 2019), antigen presentation (Watts, 2012), disease development (Nishino et al., 2000; Ballabio and Gieselimann, 2009; Appelqvist et al., 2013; Alessandrini et al., 2017) and metabolic homeostasis (Schneede et al., 2011; Lim and Zoncu, 2016; Gu et al., 2021). In addition, lysosomes contain not only a number of soluble acid hydrolases, but also plenty of integral lysosomal membrane proteins (Wilke et al., 2012). Among these proteins, lysosome-associated membrane proteins (lamps) are the major ones, accounting for nearly half of the total lysosomal membrane proteins (Eskelinen, 2006; Rajapakshe et al., 2015; Lee et al., 2016). And these proteins are involved in a variety of complex tasks, including the acidification of the lysosomal lumen, lysosomal trafficking, autophagosome-lysosome membrane fusion, exocytosis, and transport of degraded products to the cytoplasm (Saftig and Klumperman, 2009; Huang et al., 2017).

The *lamps* gene family consists of five members, *lamp1* (lgp120, CD107a) and *lamp2* (lgp110, CD107b, LGP96), *lamp3* (CD63, CD208, DC-LAMP, TSC403), *lamp4* (CD68, macrosialin) and *lamp5* (BAD-lamp) (Alessandrini et al., 2017). It has been reported that *lamp1*-deficient mice are fertile and viable (Andrejewski et al., 1999), but *lamp2* deficiency in mammals, such as in the mice or humans, leads to postnatal mortality (Tanaka et al., 2000), Danon disease (Nadeau et al., 2008), fatal cardiomyopathy and myopathy (Tanaka et al., 2000; Stypmann et al., 2006) due to a massive accumulation of autophagic vacuoles in many tissues, including skeletal muscle, liver, spleen and kidney (Tanaka et al., 2000). Previous studies have shown the function of *lamp3*, *lamp4* and *lamp5* in the human immune response, for example, *lamp3* is not only involved in recruited to *Salmonella* pathogens for intracellular proliferation (Lee et al., 2016), but overexpression *lamp3* induced lysosomal membrane permeabilization and contributes to cell death in human salivary glands (Tanaka et al., 2022; Nakamura et al., 2023b). The *lamp4* gene may have the capacity of negative regulatory functions in antigen presentation processing (Song et al., 2011). In addition, one study has shown that *lamp5* is an essential regulator of inflammatory-signaling (Lee et al., 2014). Although different isoforms of *lamp* have been found, and they have different functions in mammals. Few studies have been conducted in teleosts, which urgently need to explore the function of these genes.

The *lamp* families are highly evolutionary conserved. Structurally speaking, all lamps belong to the type I transmembrane proteins and are composed of a large, highly glycosylated luminal ectodomain with multiple N- and O-glycosylation sites (Dominguez-Bautista et al., 2015), which protects them from the action of proteolytic degradation in lysosomes (Baba et al., 2020), and a transmembrane domain, followed by a short C-terminal cytoplasmic tail (Wilke et al., 2012; Terasawa et al., 2016). In addition, the C-terminal cytoplasmic tail has tyrosine-based sorting signal motifs (-G-Y-X-X-hydrophobic residue, X, an amino acid), consisting of 11 amino acid residues that function through adaptor proteins that are recognized to be involved in trafficking of membrane vesicle coating between the *trans*-Golgi network and the lysosomal membrane (Baba et al., 2020; Sakane and Akasaki, 2018).

To date, *lamp* gene families have been isolated and identified from several animal species, including mouse (*lamp1* and *lamp2*) (Hughes and August, 1981), human ((*lamp1* and *lamp2*) (Fukuda et al., 1988), *lamp3* (Salaun et al., 2003), *lamp4* (Holness and Simmons, 1993) and *lamp5* (Defays et al., 2011)) and bird (*lamp3*) (Wu et al., 2010). In fish, however, the *lamp* genes have received far too little attention in the literature. Only four orthologs of mammalian *lamp* genes, including *lamp1*, *lamp2*, *lamp3* and *lamp4* have been characterized in zebrafish (*Danio rerio*) (Meireles et al., 2018; Iyer et al., 2022), *lamp1* was identified in japanese flounder (*Paralichthys olivaceus*) (Rondón-Barragán et al., 2017), *lamp3* was isolated from rainbow trout (*Oncorhynchus mykiss*) (Johansson et al., 2012) and *lamp4* was cloned from bluntnose black bream (*Megalobrama amblycephala*) (Cui et al., 2022). And the above results showed that *lamp* family genes play a vital immune role in teleosts.

It is well known that the fish growth and basic physiological progress are influenced by extrinsic and intrinsic factors, such as, temperature, pH, salinity, dissolved oxygen, size, age, reproductive or nutritional status (Reinecke, 2010; Fan et al., 2019; Song et al., 2019; Alfonso et al., 2021; Zengin, 2021; Liu et al., 2023). In particular, starvation stress is considered to be one of the most important intrinsic factors that not only alters the immune function (Tran et al., 2018; Song et al., 2019; Liao et al., 2021), but also changes basic energy metabolism in teleosts (Dai, W. et al., 2018; Dar et al., 2019; Florescu Gune et al., 2019; Martin et al., 2010). For instance, in *Sinibrama taeniatus*, the innate immune parameters were increased, but the adaptive immune index was declined after short period of starvation (Shi et al., 2023). In addition, starvation changes the plasma glucose level and increased lysozyme levels in *Anguilla anguilla* (Caruso et al., 2010). Furthermore, a series of studies have found that nutrient deprivation stress also induceed the autophagy in teleosts (Yabu and Yamashita, 2008; Yabu et al., 2012). For examples, many scholars indicated that the autophagosome formation was observed in the skeletal muscle and intestine of juvenile Chinese Perch (*Siniperca chuatsi*) after a short-term starvation (Wu et al., 2020; Wu et al., 2022; Pan et al., 2022). Fan and others (2020) demonstrated that nutrient deprivation induced autophagosome formation in the zebrafish embryonic fibroblast cells. Meanwhile, plenty of autophagy-related genes were also induced (Wu et al., 2022). For instance, previous studies have shown that the transcript levels of autophagy-related genes (*LC3B*, *gabarapl1*, *atg12l*, *atg4b*) were significantly induced by 14days fasting or serum deprivation of rainbow trout myocytes (Seiliez et al., 2010). Similarly, Wu et al. found that the expression of autophagy-related genes (*Atg4b/c/d*, *Becn1*, *bnip3*, *LC3a*, *LC3b*, *Gabarapl1*, and *Atp6v1d*) was significantly increased in muscle after treatment with nutrient deprivation in Chinese perch (Wu et al., 2020; Wu et al., 2022). Other studies have shown that nutrient restriction greatly increased the expression of Atg genes (*atg4*, *atg9*, *atg12*, *lc3*, *gabarap* and *becn1*) in gill epithelial cells of *O. mykiss* (*Walbaum*) (Balmori-Cedeño et al., 2019). A recent study reported that starvation stimulates *lamp1* expression in zebrafish embryonic fibroblast cells (Fan et al., 2020). However, further research and exploration are needed to study the *lamp* family genes under food-restricted conditions in teleosts.

Largemouth bass, *Micropterus salmoides*, belongs to family Perciformes, order Centrarchidae, and is one of the most important economic carnivorous fish species in China (Hu et al., 2022). It is quite popular with customers due to its lack of intermuscular bone, tasty flesh and high nutritional value (Zhu et al., 2022). However, like most of fish species, largemouth bass often suffered from food shortage and/or starvation throughout its life history due to the seasonal variations, reproduction, breeding migration and overwintering (Zou et al., 2023). To cope with the above serious problem, the autophagy-lysosomal pathway is usually initiated in fish (Belghit et al., 2014; Séité et al., 2018; Lu et al., 2019). However, few studies have focused on the effect of starvation on the lysosomal membrane proteins in largemouth bass.

In the present study, the full-length coding sequences of three lamp genes (*lamp1*, *lamp2*, *lamp3*), were isolated and characterized from the largemouth bass. Subsequently, the tissue distribution patterns of these *lamp* genes were measured to investigate their properties and functions. Meanwhile, we also determined their transcription in response to different nutritional status. Our results may provide new insights for a better understanding of the function of *lamp* genes in the responses of fish species to food deprivation and/or starvation.

## 2 Materials and methods

### 2.1 Experimental animals

In the present study, healthy largemouth bass (*M*. *salmoides*, mean weight: 30 ± 2.3 g, mean ± SEM) were obtained from a fish farm located in Jingzhou, Hubei Province, PR China and then transferred to an indoor recirculating water system (experimental aquarium 300 L) for further experiments. Fish were acclimated for 2 weeks and fed twice daily (09: 00 a.m. and 04: 30 p.m.) with commercial floating spherical food (Fuxing (Xiamen) biological feed Co., Ltd, China) at 3% of fish weight, faeces were siphoned off from the bottom of the experimental tank after 1 h of feeding, and the water was changed daily. During the acclimation and experimental periods, the water was treated with sand-filtered, the temperature was maintained at 23°C–25°C, in addition, the dissolved oxygen in the plastic tanks was maintained at a near saturation, the pH was 7.2–7.7, and the light/darkness photoperiod was 12h/12 h.

### 2.2 RNA extraction and cDNA synthesis

Total RNA was extracted from *M. salmoides* liver stored at −80°C, using Total RNA Kit I (R6731, Omega, Connecticut, United States of America) according to the manufacturer’s protocols. The concentration of RNA was then determined using spectrophotometer (Nanodrop 2000; Thermo Scientific, Wilmington, United States of America) and the integrity of the RNA was evaluated using a denaturing agarose gel (1%). Subsequently, RNA was reverse transcribed into cDNA using dsDNase (Monad, Suzhou, China) according to the operating instructions of MonScript RTIII Super Mix. The reverse transcription programme was carried out at 50°C for 15 min, followed by 85°C for 5 min, and holding at 4°C. The synthesised cDNA templates were stored at - 20°C for further study.

### 2.3 Gene cloning of the Mslamp gene

The potential sequence encoding *Mslamp* was obtained using the offline BLAST tool search against the largemouth bass genome database (Sun et al., 2021) and our transcriptome data (not shown) based on the zebrafish sequence (Genbank number: NM_001326532.1 (*lamp1*); NM_001013533.1 (*lamp2*); XM_021473556.1 (*lamp3*)); yellow perch (Genbank number: XM_028571831 (*lamp1*); XM_028589168.1 (*lamp2*); XM_028587782.1 (*lamp3*)) and large yellow croaker (Genbank number: XM_027281519.1 (*lamp1*); XM_027277608.1 (*lamp2*); XM_010736614.3 (*lamp3*)). To amplify the full-length open reading frames (ORF) of the largemouth bass *lamp* genes, specific primers pairs were designed (Table 1), and PCR amplification was performed using the following reactions: initial denaturation stage at 95°C for 3 min, 30 cycles of denaturation at 95°C for 30 s, annealing at 60°C for 30 s, elongation stage at 72°C for 60 s; and a final extension stage 72°C for 5 min. The PCR products were purified using an AxyPrep™ gel extraction kit (Axygen, United States of America) and then sequenced at Tsingke (Beijing, China).

**TABLE 1:** Primer pairs used for molecular cloning and quantitative real-time PCR.

Primers	Sequence (5′–3′)
*lamp1-01F*	CCCCCTTCTCTTTCCTCT
*lamp1-01R*	ATC​AGC​CAT​ACA​TTC​GCT​T
*Lamp1-02-F*	ACTCTCTCACGCTTTGGC
*Lamp1-02-R*	AGCATCTGGTCTTGGTCC
*Lamp1-03-F*	AACTCCACGAGCAACAAG
*Lamp1-03-R*	AAG​GAC​TAA​AGA​CAA​ACA​GC
*Lamp2-01-F*	CAGTCGGCGGCGGTAGA
*Lamp2-01-R*	GGTGTGGGGAGGGTGGG
*Lamp2-02-F*	TTACCCACAACCCCTAC
*Lamp2-02-R*	ATGAGAATCAAGCCAGC
*Lamp2-03-F*	CGGAGGGGAACACCAAC
*Lamp2-03-R*	GACACCCAGGGACCAGG
*Lamp3-01-F*	TAC​CAT​TAT​CTG​TCC​ACT​C
*Lamp3-01-R*	ATA​CCA​ACT​TTT​CTT​CTT​TT
*Lamp3-02-F*	TTT​TTT​TCC​TGG​CTG​CTA​T
*Lamp3-02-R*	CCT​TGT​CTG​CGT​CTG​TCT​T
*Lamp3-03-F*	AGT​ATG​GAA​AAG​AGG​TGG​A
*Lamp3-03-R*	AAC​AAA​TAA​ATG​TAA​AAG​C
*Lamp1-qF*	TCACTGTGGTGTCGTCGT
*Lamp1-qR*	ATCTGCCATACATTCGCT
*Lamp2-qF*	TCAGCAACGGCACCAAGA
*Lamp2-qR*	ACACCAATCCGCAGACCA
*Lamp3-qF*	ATCCAGCCTGCCTCTAAC
*Lamp3-qR*	ATTCCCAACAGCGTTTTT
*LC3-qF*	AGC​ACC​CCA​ACA​AGA​TAC​C
*LC3-qR*	CGT​TCA​TAC​ACC​TCG​CAG​A
*beclin-1qF*	AAAAAGGCAAGATCGAGG
*beclin-1qR*	TCA​GGT​TGG​TGA​GCA​TAA​A
*β-actin-qF*	TCC​TCG​GTA​TGG​AGT​CTT​G
*β-actin -qR*	GTCAGCGATTCCAGGGTA

### 2.4 Sequence analysis

Potential coding sequences were confirmed using the online ORF Finder (https://www.ncbi.nlm.nih.gov/orffinder). The deduced protein sequence was translated using DNAMAN 9.0 (Lynnon Biosoft, San Ramon, CA, United States of America). Molecular weight (MW) and theoretical isoelectric point (pI) were calculated using the ExPASy compute pI/Mw tool (https://web.expasy.org/compute_pi/). Signal peptide cleavage sites of largemouth bass lamps were predicted using SignalP-5.0 Server (https://services.healthtech.dtu.dk/service.php?SignalP-5.0). The putative protein domain features were predicted via Simple Modular Architecture Research Tool (SMART) (http://smart.embl-heidelberg.de/). Potential transmembrane regions were predicted by TMHMM (https://services.healthtech.dtu.dk/service.php?TMHMM-2.0). Amino acid sequence similarity searches of other fish lamps were performed in the National Center for Biotechnology Information (NCBI) (http://www.ncbi.nlm.nih.gov/) and the Ensemble databases (http://asia.ensembl.org/index.html). Multiple protein sequences alignment of lamp proteins was conducted using Clustal W multiple alignment program (http://www.ch.embnet.org/software/ClustalW.html). The putative N-linked glycosylation sites and O-glycosylation sites were predicted by NetNGlyc-1.0 (https://services.healthtech.dtu.dk/service.php?NetNGlyc-1.0) and NetNGlyc-4.0 (https://services.healthtech.dtu.dk/service.php?NetOGlyc-4.0), respectively. NetPhos-3.1 (https://services.healthtech.dtu.dk/service.php?NetPhos-3.1) was employed to predict putative C-terminal phosphorylation sites. The percentage of similarity and identity of lamp proteins from other species was calculated by Bioedit. The online SWISS-MODEL tool (https://www.swissmodel.expasy.org/) and phyre^2^ (http://www.sbg.bio.ic.ac.uk/phyre2/html/page.cgi?id=index) were applied to predict the 3D-structure of selected lamp proteins.

### 2.5 Syntenic and gene structures analysis

To further investigate the identification of *Mslamp*, the synteny and gene structures were implemented via comparative genomic survey (Tan et al., 2019; Wen et al., 2019; Hu et al., 2022). The exon/intron organization of *Mslamp* genes was predicted by FGENESH software (http://linux1.softberry.com/berry.phtml?topic=fgenes_plus&group=programs&subgroup=gfs). The genome databases of several representative vertebrate species, such as, *D. rerio* (GRCz11, Ensembl), *Homo sapiens* (GRCh38, Ensembl), *Mus musculus* (GRCm38.p6, Ensembl), *Xenopus tropicalis* (UCB_Xto_10.0, Ensembl), *Gallus gallus* (GRCg6a, Ensembl) and *Lepisosteus oculatus* (LepOcu1, Ensembl) were selected as the reference.

### 2.6 Phylogenetic analysis

To determine the evolutionary position of *Ms*LAMPs and the phylogenetic relationship with other teleost LAMPs, the phylogenetic tree was constructed by Neighbor-Joining (NJ) method based on LAMPs protein sequences using Molecular Evolutionary Genetic Analysis (MEGA 6.0) according to the methods described in the literature (Wen et al., 2019). Briefly, the LAMPs protein sequences of several vertebrate species were retrieved both from the NCBI and the Ensemble databases (Supplementary Table S3), and then these selected amino acid sequences of LAMPs were used to perform multiple protein sequence alignment in Clustal multiple software. Subsequently, the aligned amino acid dataset was used to construct a phylogenetic tree using NJ approach of MEGA 6.0 with the JTT matrix-based model. The robustness of the trees was assessed by 1,000 bootstrapping iterations. In addition, the lamprey species was considered as an outgroup.

### 2.7 The tissue distribution of Mslamp1, lamp2 and lamp3

To study the tissue distribution of three *Mslamp1*, *lamp2* and *lamp3,* six healthy fish were randomly selected, and anesthetized with MS-222 (10 mg/L^-1^). Various selected tissues, including liver, brain, gill, heart, muscle, spleen, kidney, stomach, adipose and intestine were isolated, and then rapidly frozen in liquid nitrogen and stored at - 80°C for tissue expression analysis.

### 2.8 Treatment with different feeding status

To investigate the expression of *lamp1*, *lamp2* and *lamp3* in *M. salmoides* after food deprivation (2-week) and refeeding experiment, 135 healthy fish (30 ± 2.5 g, mean ± SEM) were randomly divided into three experimental groups: control group, fasted group and refeeding group, and each group contained three tanks (15 individuals/tank). Fish were acclimatized and fed under the same conditions (2.1) for 2 weeks prior to the experiment. The fed group was kept on the same feeding strategy and the fasted group was not fed throughout all the treatment period. In the refeeding group, after 2 weeks of fasting, refeeding was started for 2 days with the same feeding ration as in the control group. Liver, kidney and spleen were selected for analysis as they play a crucial metabolic and physiological role under the stress background information (Hu et al., 2022). Fish were randomly selected and euthanised with tricaine methanesulfonate (MS-222, 10 mg/L) for sampling. For each experimental group, liver, kidney and spleen samples were collected from 18 fish (6 fish per tank) and frozen in liquid nitrogen and subsequently stored in - 80°C.

### 2.9 Real-time fluorescence qPCR analysis

Total RNA was extracted from samples, and first strand cDNA synthesis was conducted as described above (2.2). The specific primers (Table 1), synthesized by Tsingke (Beijing, China), were designed based on the full-length coding sequences of the largemouth bass *lamp* genes, and the specific primers were verified by PCR and the amplification determined by 1% agarose gel electrophoresis. The cDNA was diluted five times with ddH_2_O and used as a template. In the present study, MonAmp™ ChemoHS qPCR Mix (Monad, China) and LineGene 9,600 Plus (Bioer, China) were used to monitor the expression level of *lamp* genes in different tissues and after the feeding status treatment. The RT-qPCR reaction mixture consisted of 2 μL of cDNA, 10 μL of MonAmp™ ChemoHS qPCR Mix (Monad, China) and 0.4 μL of gene-specific primer in a total volume of 20 μL. The amplification conditions were as follows: preincubation at 95°C for 5 min, followed by 40 cycles of denaturing at 95°C for 10s, annealing at 60°C for 10s, and extension stage at 72°C for 30s. In this study, *β-actin* was used as an internal reference gene to analyze the relative expression of *lamps* gene (Hu et al., 2022). All reactions were performed in duplicate and each reaction was verified by the melting curves, which showed a single peak specific for the target genes. The relative transcript levels of *lamps* genes were calculated using the 2^−ΔΔCT^ method (Pfaffl, 2001).

### 2.10 Statistical analysis

Statistics were performed using the SPSS 18.0 (IBM, Armonk, NY, United States of America). Graphs were drawn by Prism 8.0 (Graph Pad Software Inc., San Diego, United States of America). Data are expressed as mean ± SEM (standard error of mean). One-way analysis of variance (ANOVA) was used to assess the significant differences followed by Duncan’s multiple range tests. A difference was considered significant if *p* < 0.05.

## 3 Results

### 3.1 Molecular characteristics of the lamp genes from largemouth bass

**FIGURE 1:**
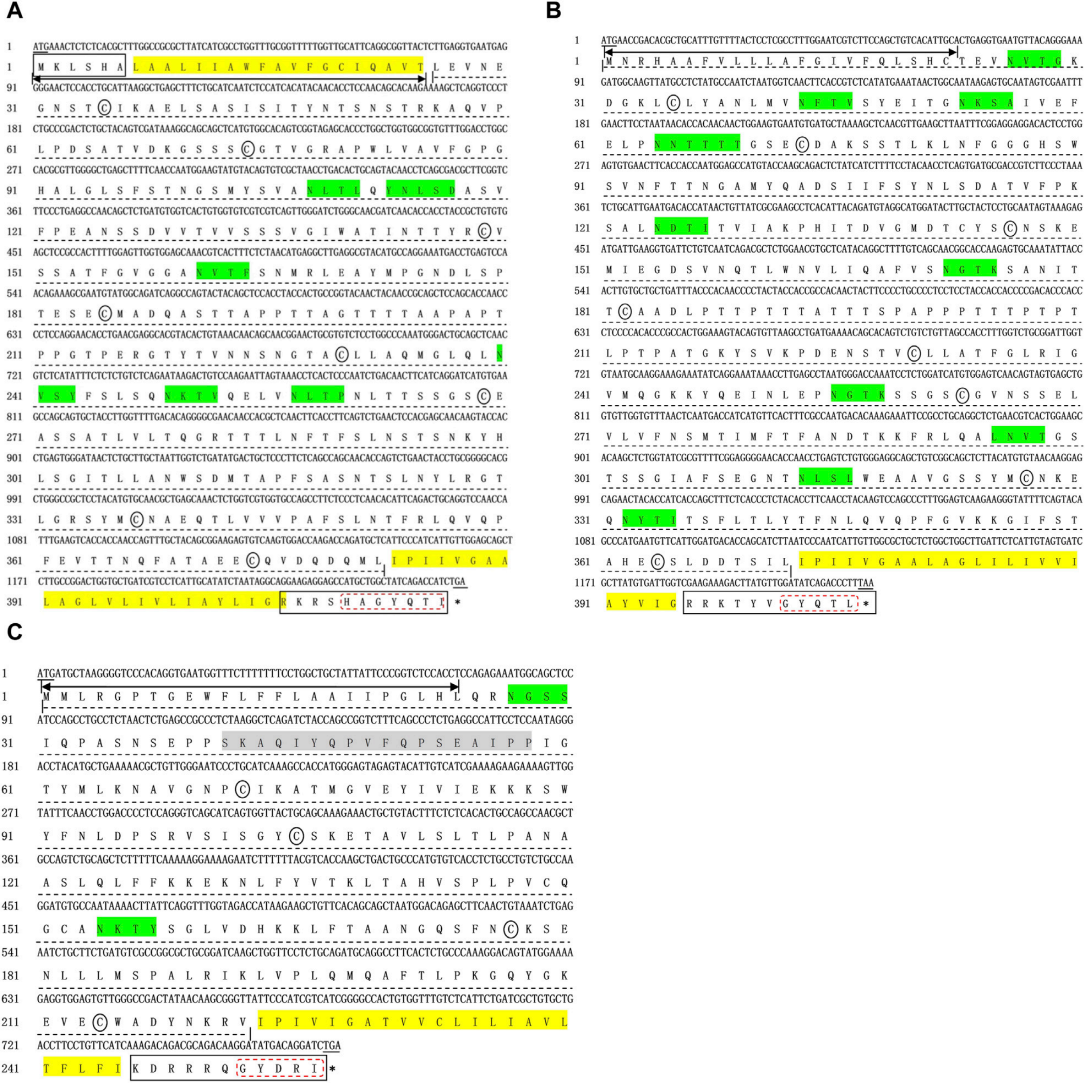
Complete coding sequences and deduced protein sequences of the *lamp1* **(A)**, *lamp2* **(B)**, and *lamp3* **(C)** gene in Largemouth bass (*Micropterus salmoides*). The numbers denoting the positions of nucleotide and amino acid were marked on the left. Underlines represent the initiation codon and termination codon respectively, and stop codon was shown with a black asterisk (*). Two-way arrow indicates the singal peptide. The transmembrane domains are marked by yellow frame. Black solid box and dotted line shows the cytoplasimic domain and extra-cellular domain, respectively. The conserved cysteines are indicated with black ellipse. The conserved C-terminal tyrosine-based lysosomal targeting motif (Tyr-X-X-hydrophobic residue) is showed in red dotted frame. The potential N-glycosylation sites are indicated with green solid frame.

**FIGURE 2:**
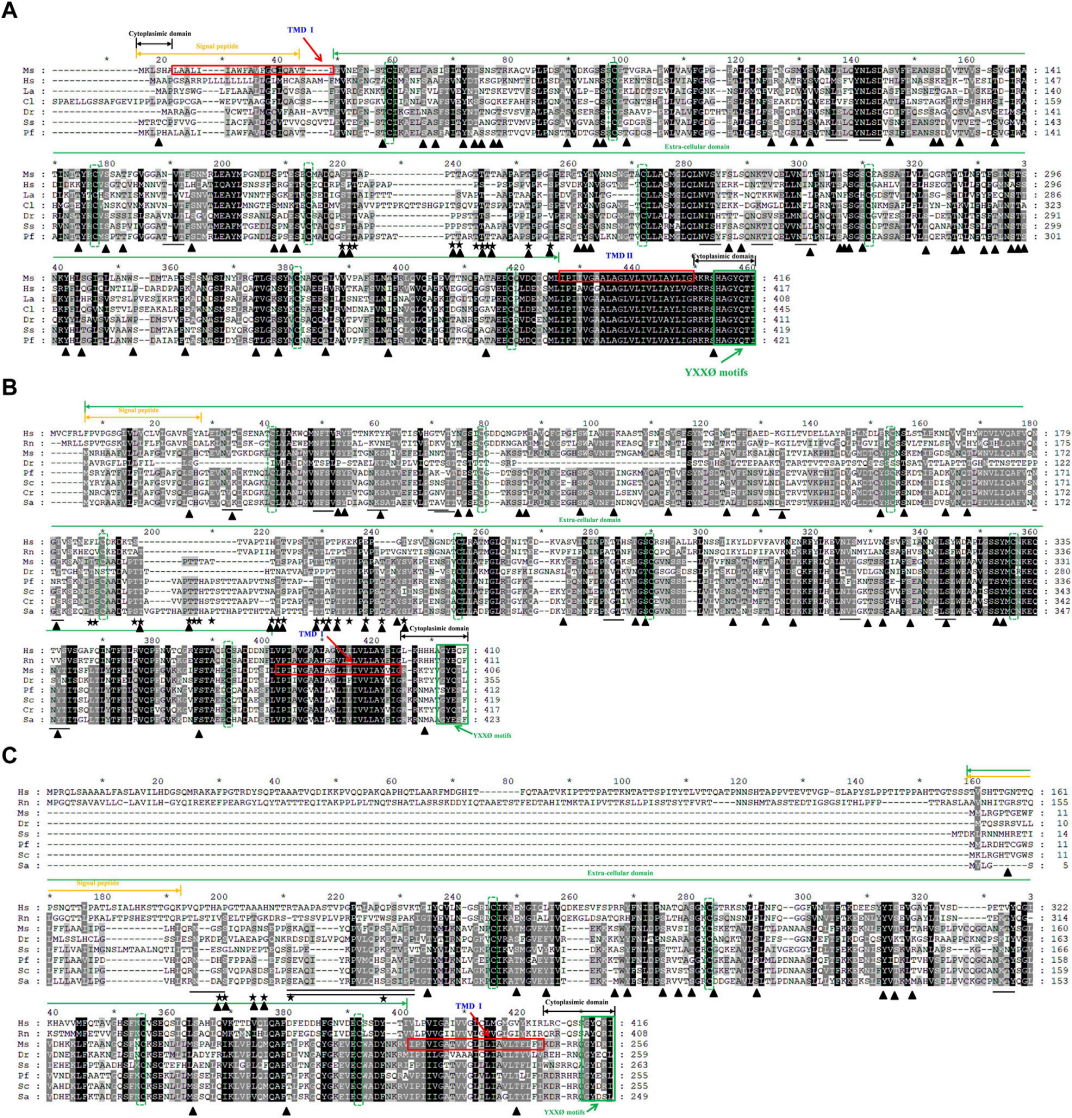
Multiple amino acid sequence alignments of largemouth bass LAMP1 **(A)**, LAMP2 **(B)** and LAMP3 **(C)** were compared with those of mammals and other vertebrates. The multiple alignments were conducted using the Clustal X program (version 1.8) and shaded using Genedoc (version 2.7). The conserved amino acid residues are in black, similar amino acid residues are in dark gray. The signal peptide is indicated by the orange two-way arrow. The transmembrane domains are marked by red frame. Cytoplasimic domain and extra-cellular domain are depicted in black and green two-way arrow, respectively. The conserved cysteines are indicated with green dotted frame. The potential N-glycosylation sites in Ms-LAMP are underlined. The potential O-glycosylation sites are marked by star. The potential phosphorylation sites are highlighted in black triangle. The conserved serine/proline-rich region is underlined with a double line below. The conserved C- terminal tyrosine-based lysosomal targeting motif (YXXØ) is showed in green solid frame.

**FIGURE 3:**
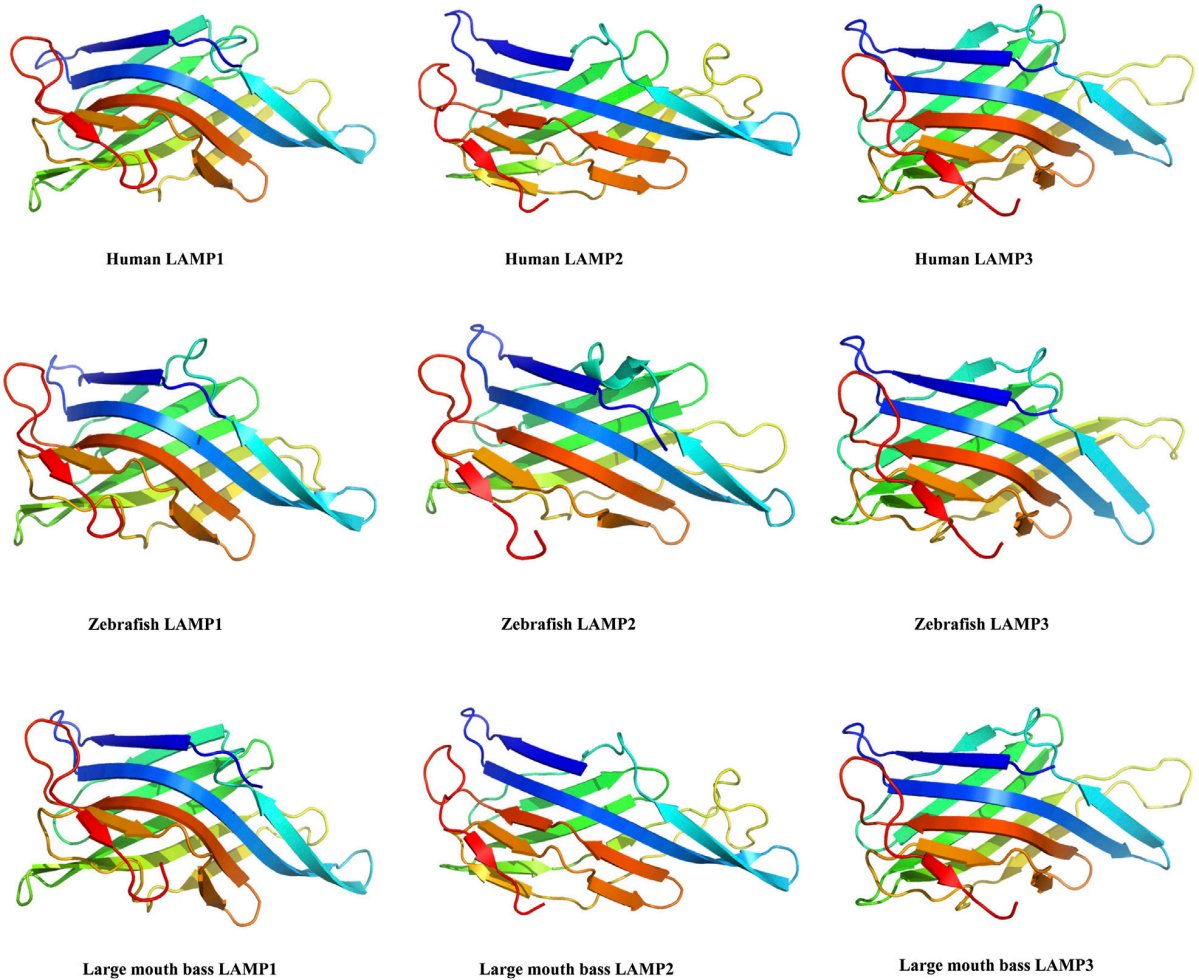
The 3D-structure of the largemouth bass *lamp1-3* genes were predicted by AWISS-MODEL online program and phyre^2^ 73 tool.

To capture the full-length coding sequence of the *Mslamps* genes, pairs of specific primers were designed (Table 1). Subsequently, the verified fragments of sequence were assembled into individual complete ORF sequence using DNAMAN software, respectively. Overall, the sequence characteristics of Ms*lamp1*-*3* were summarized in Table 2. The ORF for *lamp1*, *lamp2* and *lamp3* have a length of 1251bp, 1224bp and 771bp, encoding for 416 amino acid (aa), 407 aa and 256 aa residues, respectively. The deduced molecular mass (kDa)/isoelectric point of *lamp1*, *lamp2* and *lamp3* were calculated to be 104,993 kDa/4.97, 43,546 kDa/5.98 and 28,482 kDa/9.53, respectively (Table 2). In addition, the signaling peptide, transmembrane regions, potential N-glycosylation sites, predicted O-glycosylation and predicted phosphorylation sites of *lamp1*, *lamp2* and *lamp3* were 1/2/6/11/64, 1/1/11/19/47, 1/1/2/6/21, respectively (Figures 1–3; Table 2). Finally, the verified nucleotide sequences of *lamp1*, *lamp2* and *lamp3* were submitted and deposited in GenBank under accession numbers: OR587856; OR587857 and OR587858, respectively.

**TABLE 2:** The basic molecular information of three Lamps genes from largemouth bass.

Iterms	Gene		
	*Lamp1*	*Lamp2*	*Lamp3*
ORF (bp)	1,251	1,221	771
Length of amino acid (aa)	416	406	256
Molecular weight (kDa)	104,993	43,546	28,482
Isoelectric point pI	4.97	5.98	9.53
Signal peptide	1	1	1
Transmembrane region	2	1	1
Accession no.	OR587856	OR587857	OR587858

### 3.2 Multiple alignments and 3-D structure prediction of largemouth bass lamps

Multiple alignment of the protein sequences revealed a highly degree of conservation of the LAMP 1-3 protein sequences across vertebrates (Figure 2). In total, these have the typical features of a type I transmembrane protein, i.e., a signal peptide, a long extracellular domain, a hydrophobic transmembrane domain and a short cytoplasmic domain, with the exception that *Ms*LAMP1 contains a short cytoplasmic domain in the N-terminal region. Furthermore, all *Ms*LAMP family proteins have a conserved tyrosine-based motif (TyrX-X-hydrophobic residue or YXXØ sorting signal) (412-416 aa for LAMP1 (Figure 2A), 402-406 aa for LAMP2 (Figure 2B) and 252-256 aa for LAMP3 (Figure 2C)) in the corresponding of short C-terminal cytoplasmic domain, which is required for lysosomal targeting (Guarnieri et al., 1993; Wu et al., 2010). Sequence alignment analysis further revealed that both of *Ms*LAMP1 and *Ms*LAMP2 possess eight conserved cysteine residues, but the *Ms*LAMP3 has only four conserved cysteine residues with the potential to form four and two disulfide bridges, respectively (Rondón-Barragán et al., 2017). In addition, similar to other LAMP family members, a proline/serine-rich or proline/threonine region was also found between the luminal and proximal domains in LAMP1-3 protein sequences. To our surprise, the N-terminal region of LAMP3 was absent in all fish sequences (Figure 2C) (Johansson et al., 2012).

Amino acid homology of LAMP family members was performed using DNASTAR Lasergene software, with the results showing that all amino acid sequence identities between *Ms*LAMP1, *Ms*LAMP2, *Ms*LAMP3, and other teleosts ranged from 40.7% to 99% (Supplementary Tables S4–S6). *Ms*LAMP1 shares a higher identity with its counterparts from teleosts such as *Micropterus dolomieu* (99%), followed by those from *Sparus aurata* (88.9%), *Epinephelus lanceolatus* (88%), *Perca flavescens* (85.8%) and *Channa argus* (83.2%). A relatively low amino acid identity (43.5%) was observed for *Ms*LAMP1 compared to *H. sapiens* (Supplementary Table S4). A similar pattern was found for *Ms*LAMP2, which had the highest similarity identity with that of other fish species, such as: *S. chuatsi* (76%), followed by *Perca flavescens* (71.2%) and *S. aurata* (69.5%), while the low similarity of *Ms*LAMP2 with *H. sapiens* LAMP2 was 39.4% (Supplementary Table S5). In addition, *Ms*LAMP3 shared the highest identity with *S. chuatsi* (87.5%), followed by *S. aurata* (73.9%), *Perca flavescens* (73.2%), *Larimichthys crocea* (70%), and the lowest identity with *H. sapiens* (25.7%) (Supplementary Table S6). Notably, the amino acid homology of *Ms*LAMP1, *Ms*LAMP2 and *Ms*LAMP3 shared low identity with each other, i.e., the *Ms*LAMP1 shared only 37.5% and 24.7% identity with that of *Ms*LAMP2 and *Ms*LAMP3, respectively. And *Ms*LAMP2 possessed 22.6% identity with that of *Ms*LAMP3.

The three-dimensional structures of the largemouth bass LAMPs were predicted by Phyre 2. They were all modelled with 100.0% confidence by the single highest scoring template (crystallographic structures c5gv0A, 5gv3.2.A and 4akm.1.A were selected as template models, respectively). The predicted 3D structure revealed that the individual largemouth bass LAMPs and those of other vertebrates (such as human and zebrafish) share a greatly similar structure (Figure 3), which may have similar functions.

### 3.3 Phylogenetic analysis

**FIGURE 4:**
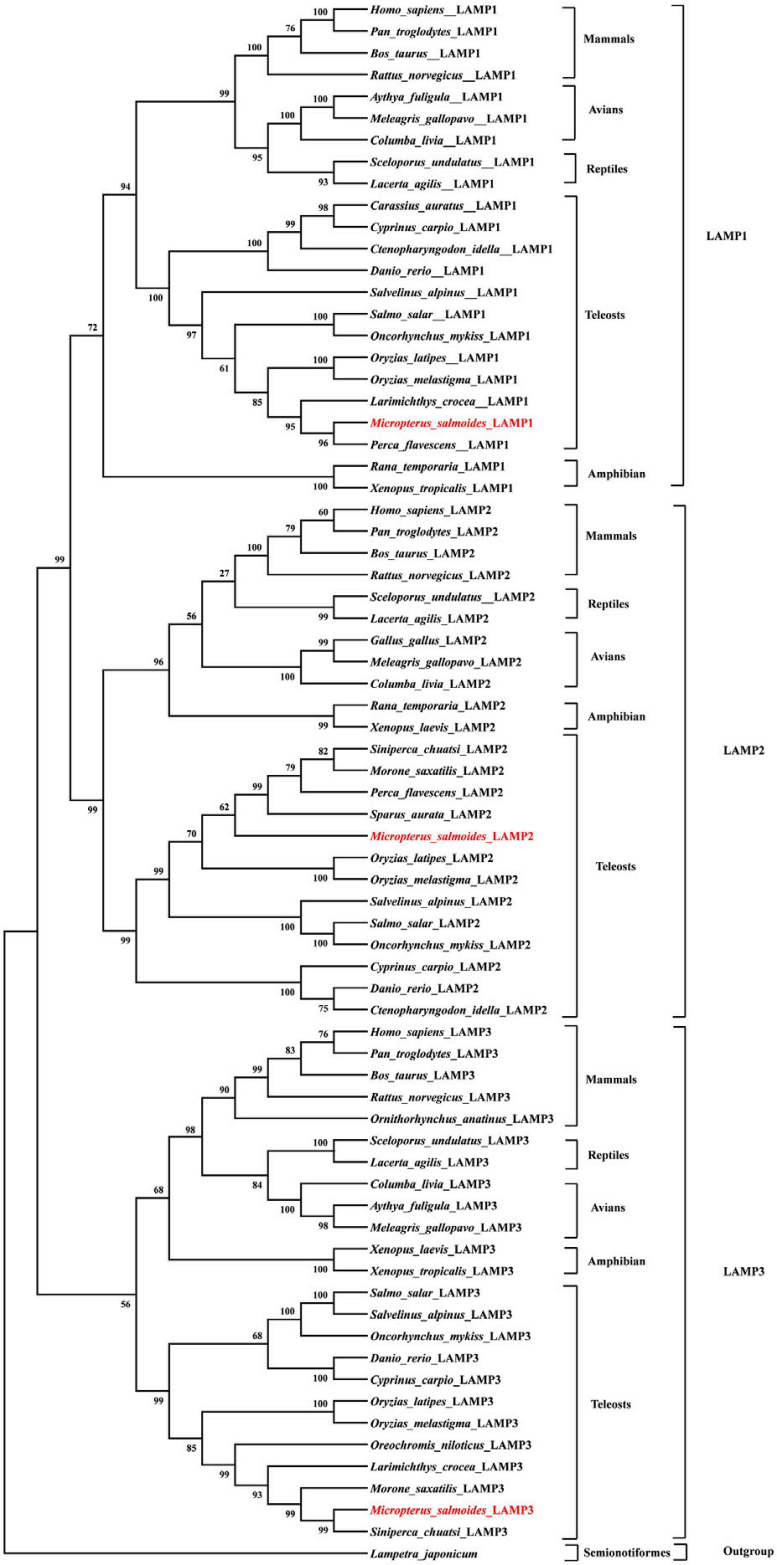
Phylogenetic tree of LAMP1, LAMP2 and LAMP3 protein sequences. The phylogenetic tree indicated the evolutionary relationship between LAMP1, LAMP2, LAMP3 proteins and the other vertebrate species. It was constructed using an a multiple alignment and the neighbor-joining method of MEGA 6.0 program. The percentage of trees in which the associated taxa clustered together, and values at the nodes represent bootstrap percentages from 1,000 replicates. The sequence of Lepisosteus oculatus (Spot gar) was used as an outgroup to root the tree.

To clarify the evolutionary phylogenetic relationships between *Ms*LAMPs and other vertebrate LAMPs, a phylogenetic tree was constructed using MEGA 6.0, with *Lampetra japonicum* as an outgroup out root the phylogenetic tree. The tree indicated that the inferred vertebrate LAMP phylogeny was divided into three major subfamilies (Figure 4). The vertebrate branch was consistent well with established taxonomic relationships. And each subfamily was composed of group mammals, avians, reptiles, amphibians and teleosts. As expected, in all clades, each LAMP was clustered with closer to the corresponding counterparts of fish than other vertebrates (mammals, amphibians, reptiles and avians). Within the fish group, we found that the *Ms*LAMP1, *Ms*LAMP2 and *Ms*LAMP3 have a close relationship with their homologues in *Perca flavescens*, *S. aurata*, and *S. chuatsi*, respectively (Figure 4).

### 3.4 Genomic structure and syntenic analysis of vertebrate lamp family genes

The DNA structure and synteny of the vertebrate lamp family genes were analyzed in different representative species based on the location of lamp in the published genomic data, such as, mammals (human and mouse), avian (chicken), amphibian (tropical clawed frog), reptiles (australian saltwater crocodile), perciformes (large yellow croaker, barramundi perch, gilthead seabream, climbing perch and amazon molly), tetraodontiformes (tetraodon and fugu), cyprinformes (zebrafish), lepisosteiformes (spotted gar) and esociformes (northern pike).

**FIGURE 5:**
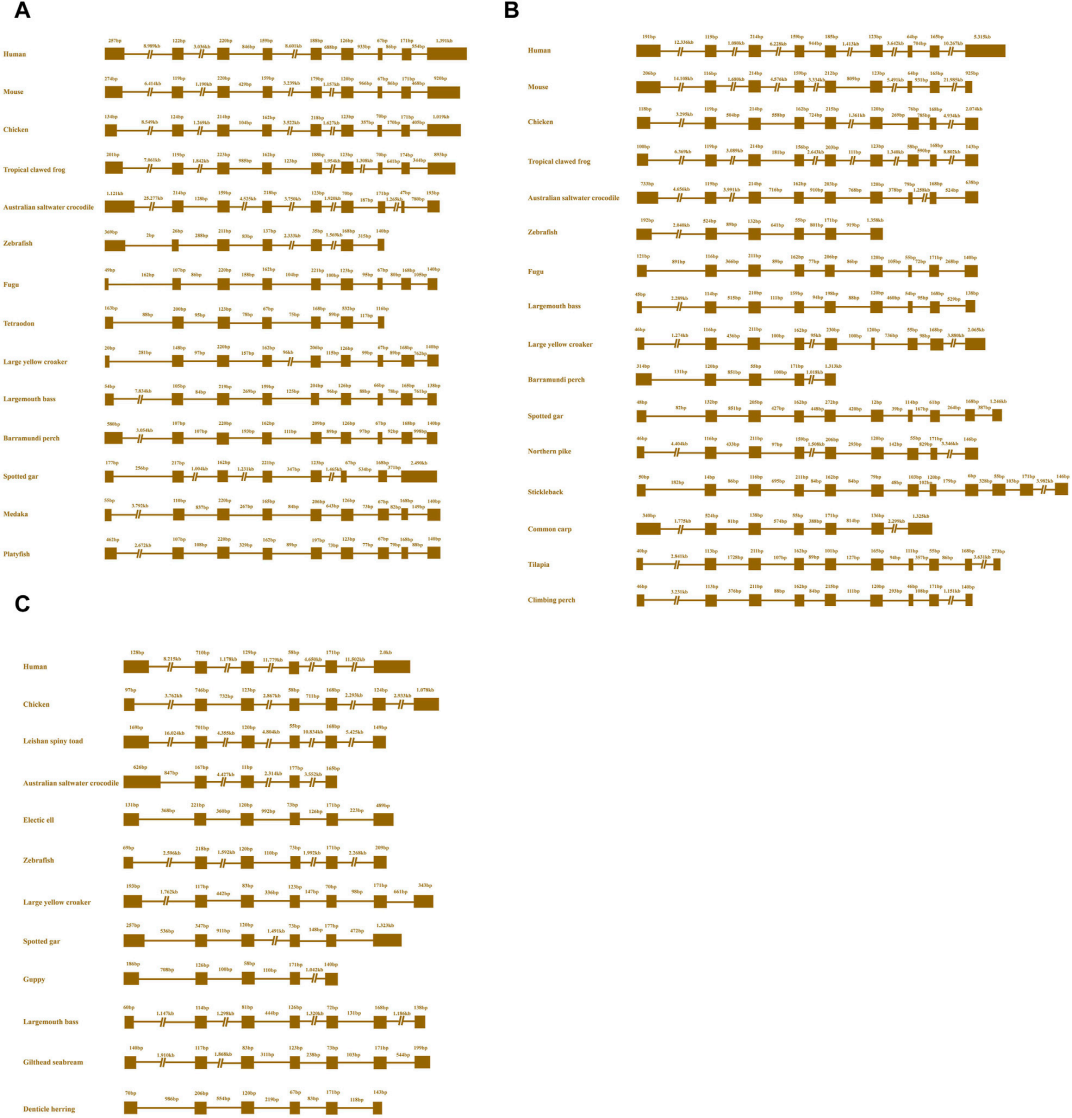
Schematic diagrams of exon/intron structural comparisons of the lamp1 **(A)**, lamp2 **(B)**, lamp3 **(C)** in various vertebrate species. The brown solid lines and brown solid box represent introns and a coding region with exons, respectively. As shown in the figure, the size of exons and introns are numbers above the colorful boxes and brown solid lines, respectively.

In silico analysis of the *lamp* gene structure revealed that both the largemouth bass *lamp1* and *lamp2* genes share a similar gene structure with most of vertebrates, containing nine exons and eight introns each, whereas largemouth bass *lamp3* gene possesses seven exons and six introns (Figure 5).

**FIGURE 6:**
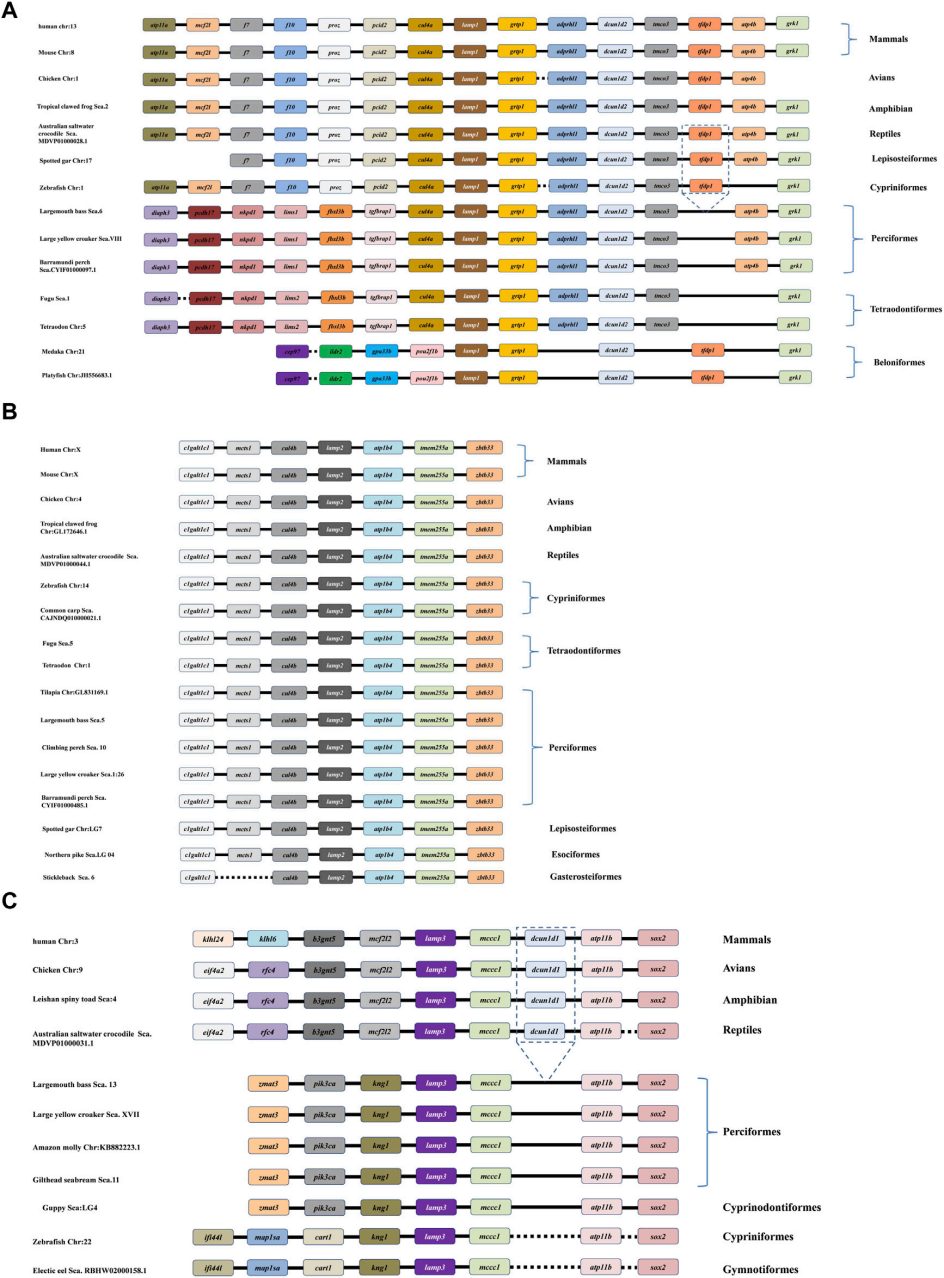
Comparative synteny analysis of *lamp1* **(A)**, *lamp2* **(B)** and *lamp3* **(C)** genes were conducted in the variety of representative vertebrate genomes. Genes and intergenic regions are showed with different colorful blocks, solid and dotted lines, respectively. As depicted in the figure, the *lamp1-3* genes have been identified in all the selected fish genomes.

Similar to the tetrapod lineage, the results showed that three *lamp* genes were presented in all representative vertebrates according to their relative locations on the genome (Figure 6). As depicted in Figure 6, from fish to human, different conserved syntenic relationships were found between *cul4a* and *grtp1* gene, *clgalt1c1* and *zbtb33* gene, *kng1* and *sox2* gene, for *lamp1*, *lamp2* and *lamp3*, respectively. We also observed that the *lamp1*, *lamp2* and *lamp3* genes are located on different chromosomes (Figure 6).

Interestingly, a specific gene cluster, *cul4a*-*lamp1*-*grtp1*-*adprhl1*-*dcun1d2*-*tmco3*, which belongs to the downstream gene of *lamp1*, was more conserved between tetrapods and several teleosts, respectively (Figure 6A). Surprisingly, the *tfdp1* gene was lost in the downstream region in perciformes (largemouth bass, barramundi perch, and large yellow croaker) and tetraodontiformes (fugu and tetraodon) compared to the corresponding loci in the other vertebrates. In addition, the different order (*pou2f1b*-*lamp1*-*grtp1*-*dcun1d2*) of the *lamp1* gene was observed in beloniformes (medaka and platyfish) (Figure 6A).

Comparison with tetrapod and fish species revealed that the *lamp2* gene has a specific cluster, *clgaltlc1*-*mcts1*-*cul4b*-*lamp2*-*atp1b4*-*tmem255a*-*zbt33*, and it showed highly conserved synteny in all representative genomes examined. However, the *mcts1* gene disappeared in the *lamp2* gene cluster in gasterosteiformes (Stickleback) (Figure 6B).

It appears that there was a greatly conserved syntenic relationship, the *lamp3*-*mccc1*-*dcun1d1*-*atp11b*-*sox2* gene cluster, among the downstream genes of vertebrate *lamp3*, although the *dcun1d1* gene was missing in all teleost species examined. In comparison, the upstream genes showed more significant differences between the tetrapods and teleosts (Figure 6C).

### 3.5 Tissue distribution of Mslamps family genes

**FIGURE 7:**
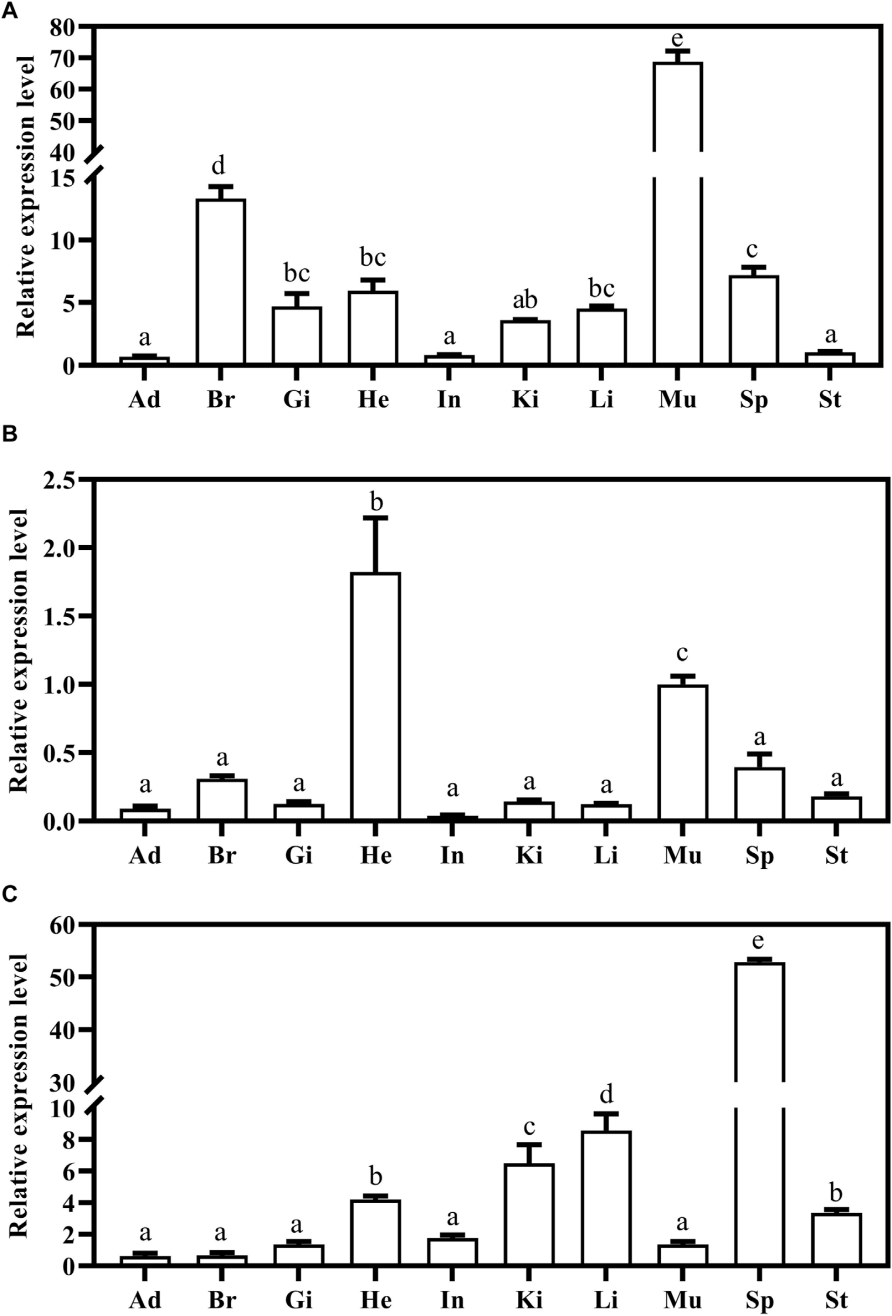
The expression of largemouth bass *lamp1* **(A)**, *lamp2* **(B)** and *lamp3* **(C)** genes in the different tissues (Ad, Adipose; Br, Brain; Gi, Gill; He, Heart; In, Intestine; Ki, Kidney; Li, Liver; Mu, Muscle; Sp, Spleen; St, Stomach) were examined by qRT-PCR. The expression value was presented as a ratio (the *lamp* mRNA level normalized to the corresponding *β -actin* gene in tissues values). Error bar represents a standard error of the mean (n = 6). Values with different letters represent statistical significance (p < 0.05).

Expression of *Mslamps* family member transcripts were detected using qRT-PCR, in ten test tissues, including adipose, brain, gill, heart, intestine, kidney, liver, muscle, spleen and stomach. Our data suggested that the *Mslamp* family members were constitutively present in all tissues examined, but at different expression levels. Among them, the *lamp1* gene exhibited highly expression level in the brain and muscle (*p* < 0.05) (Figure 7A). Similarly, the highest transcript level of the *lamp2* gene was found in the heart (*p* < 0.05) (Figure 7B). Differently, the relative expression level of *lamp3* gene was significantly higher in the spleen than that in other tissues (*p* < 0.05) (Figure 7C).

### 3.6 Expression profile of Mslamps family genes after food deprivation and refeeding treatment

**FIGURE 8:**
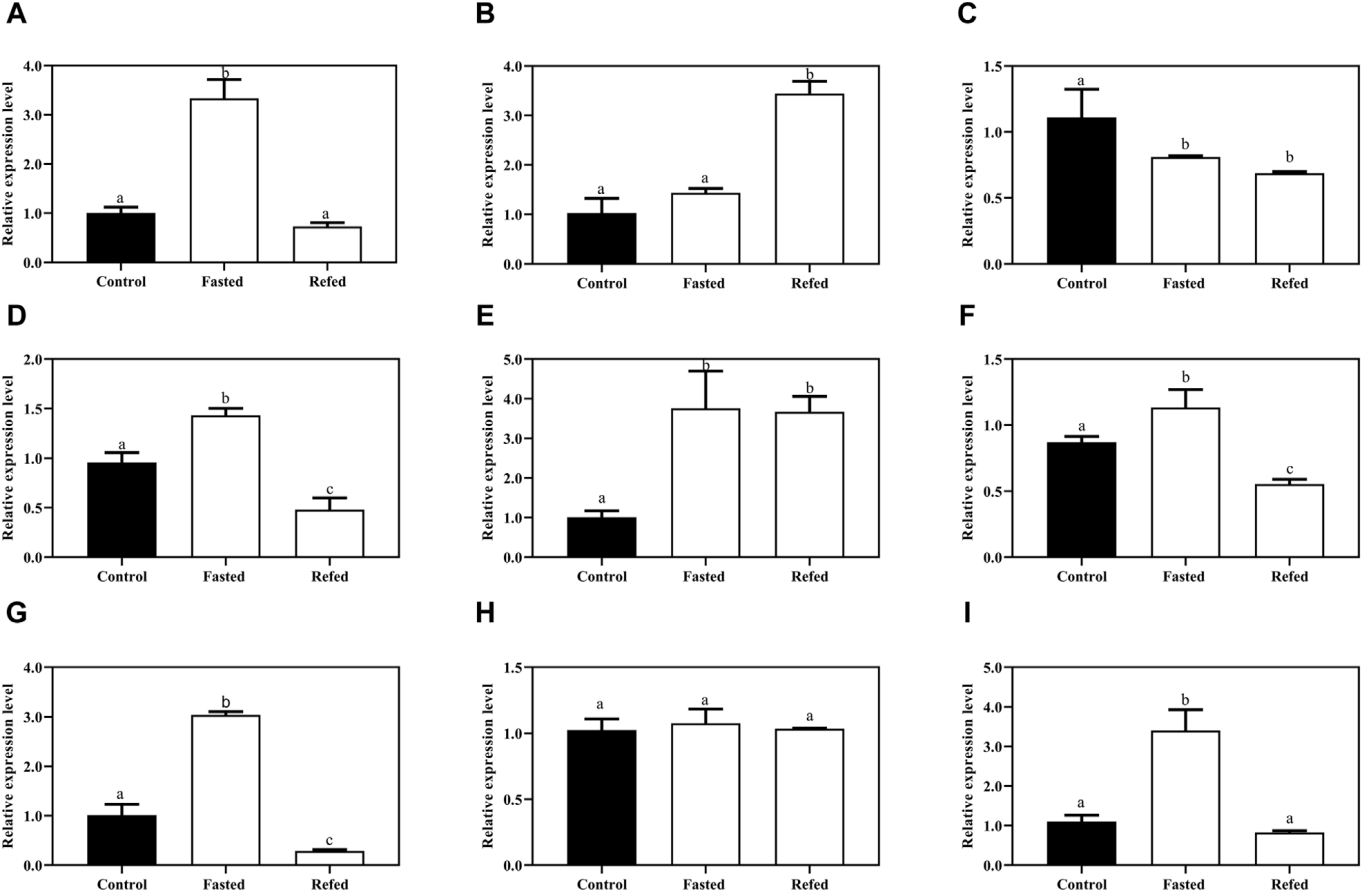
The expression levels of *lamp1-3* genes in the liver **(A,D,G)**, spleen **(B,E,H)**, kidney **(C,F,I)** of largemouth bass after long-term fasting and refeeding treatments were detected by qRT-PCR (n = 6). The expression value was presented as a ratio (the lamp mRNA level normalized to the corresponding *β -actin* gene in tissues values). Error bar represents a standard error of the mean (n = 6). Values with different letters represent statistical significance (p < 0.05).

To investigate how the *Mslamp* family members are modulated in response to starvation and refeeding regimes, we examined the different tissues (liver, spleen and kidney) during the long-term food deprivation and refeeding. As shown in Figure 8, the expression level of *lamps* appeared to be tissue specific, with the *lamp1* mRNA level in the liver significantly increased after starvation, and then it was dramatically fall down to the level of the control group after refeeding (Figure 8A). Surprisingly, the transcript level of *lamp1* in kidney was much lower in both the fasted and refed groups than that in the control group (*p* < 0.05), and no significant differences were observed between the fasted and refed groups (*p* > 0.05) (Figure 8C). In contrast, the opposite phenomenon was observed in the spleen (Figure 8B).

A similar significantly elevated *lamp2* transcription pattern was detected in both liver and kidney after fasting, whereas it was remarkably declined after refeeding compared with the control group (Figures 8D, F). In addition, the splenic *lamp2* expression level in fasted and refeeding group was much higher than that in the control group (*p* < 0.05), and no significant differences were detected between the fasted and refeeding groups (*p* > 0.05) (Figure 8E).

It was found that the expression level of *lamp3* was increased after fasting compared with the control group, and then significantly decreased to or even below the control group level after refeeding in liver and kidney (Figures 8G, I). Nevertheless, the expression of *lamp3* in the spleen was not changed among the three groups (*p* > 0.05) (Figure 8H).

**FIGURE 9:**
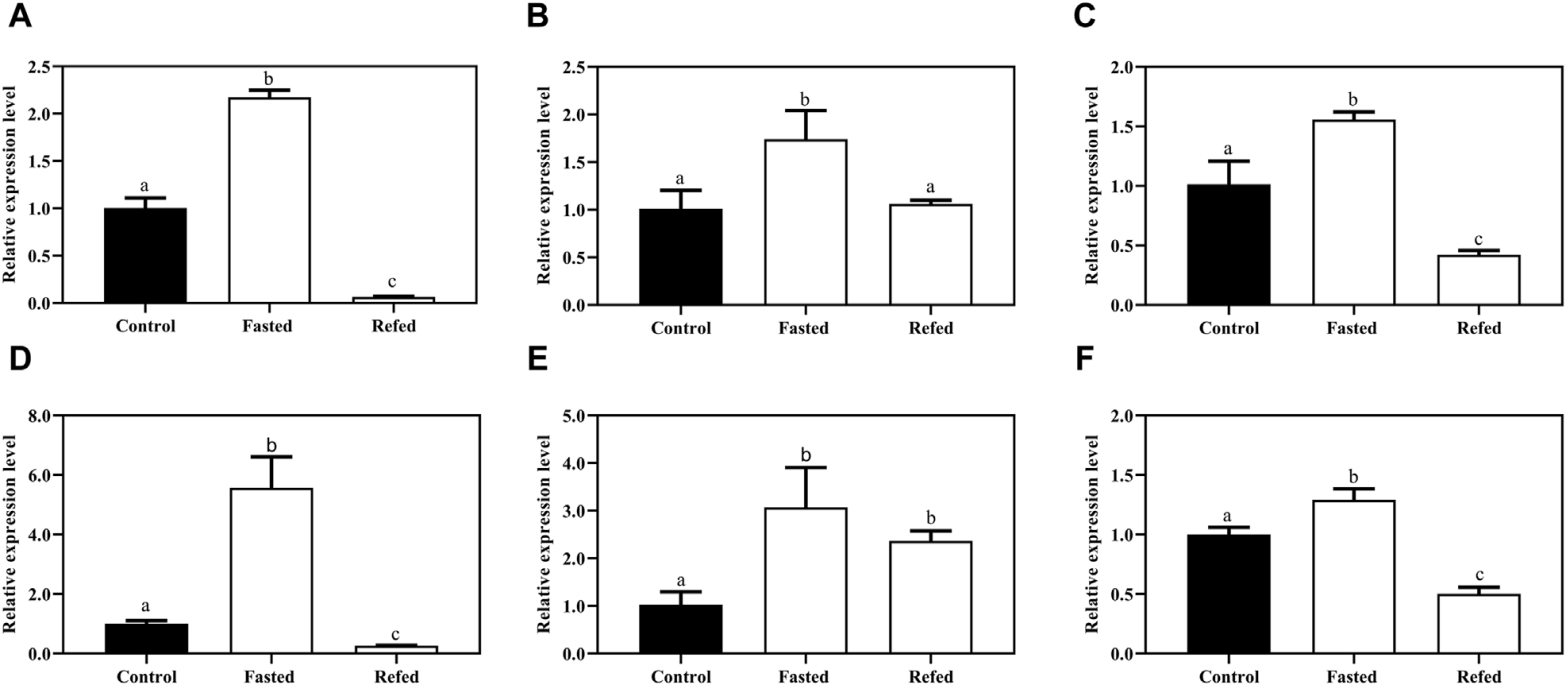
The expression levels autophagy-related genes the Beclin-1 **(A,B,C)** and MAPLC3 **(E,F,G)** of largemouth bass after long-term fasting and refeeding treatments were detected by qRT-PCR (*n* = 6). The expression value was presented as a ratio (*the lamp* mRNA level normalized to the corresponding *β -actin* gene in tissues values). Error bar represents a standard error of the mean (*n* = 6). Values with different letters represent statistical significance (*p* < 0.05).

To confirm whether or not the autophagy was induced by the starvation and refeeding strategy, in the present study, we examined the transcript levels of two known autophagosomal markers, *Beclin1* and *MAPLC3* and/or *LC3* (microtubule-associated protein1 light chain 3), after starvation and refeeding treatment in the aforementioned tissues. The results showed that *Beclin1* mRNA expression was strikingly upregulated after fasting in the tested tissues compared to the control group, and then significantly downregulated after refeeding in three tested tissues (Figures 9A–C). A similar expression pattern of *MAPLC*3 was observed in three tissues in response to different feeding statuses (Figures 9D–F).

## 4 Discussion

In the current study, three of the *lamp* family genes (*lamp1*, *lamp2* and *lamp3*) of largemouth bass (*M. salmoides*) were successfully identified and characterized. The full-length coding sequences of *lamp1*, *lamp2* and *lamp3* were 1,251, 1,221 and 771bp, and predicted to encode a protein of 416, 406 and 256 amino acids, respectively, which is consistent with previous findings, such as in japanese flounder (Bandyopadhyay et al., 2014; Rondón-Barragán et al., 2017), zebrafish (GenBank no. NM_001013533) and rainbow trout (Johansson et al., 2012), indicating that the family of *lamp1*-*3* genes may be conserved in fish. The multiple sequence alignment revealed that LAMP1-3 proteins contain a typical feature of a type I transmembrane (a signal peptide, a long extra-cellular domain, a hydrophobic transmembrane domain and a short cytoplasmic domain), protein cysteine residues and proline/threonine region, that is highly conserved among vertebrates, showing that LAMP1-3 proteins of *M. salmoides* may have the same function in other species. In addition, a conserved tyrosine-based sorting signal motif (YXXØ sorting signal) located at the carbon terminal was also observed, which is capable of targeting the lysosome (Guarnieri et al., 1993). Interestingly, the N-end of the LAMP3 protein was missing in all teleosts, which is similar to a previous report carried out in the sequences of the trout, salmon and zebrafish (Johansson et al., 2012), suggesting that the functional conservation of *lamp3* in fish.

To investigate the evolutionary history of *lamp1-3* genes in vertebrates, a phylogenetic analysis was conducted based on the protein sequences, selected from these representative species. We observed that the relationship between *M. salmoides* and other teleosts and vertebrates in the constructed phylogenetic tree is in consistent with traditional systematics (Figure 4). Meanwhile, the phylogenetic analysis showed that the LAMP1, LAMP2 and LAMP3 were clustered together with the homologous counterparts from other fishes and vertebrates, and divided into three subclades, which is in line with a previous study carried out by Johansson (Johansson et al., 2012).

In the current study, mutil-copies *lamp* (*1–3*) genes in *M. salmoides* were located on different chromosomes and possessed variable numbers of exons and introns (Figure 5), which is likely to be due to gene duplication events (Morash et al., 2010). Interestingly, the number of exons and introns of *lamp3* is much lower than that of *lamp1* and/or *lamp2*, further suggesting that these *lamp* genes may have different physiological functions in fish species. This is consistent with many studies in mammals, for instance, previous studies found that the *lamp*2-deficiency is closely associated with Danon disease, which is characterized by developmental disability, cardiomyopathy and myopathy in mammals (Malicdan et al., 2008; Nishino, I et al., 2000). Sakane and Akasaki (2018) demonstrated that *Lamp1* was involved in myoblasts differentiation. Meanwhile, *lamp3* was found to be involved in the regulation of diseases associated with hepatic lipid metabolism disorders (Liao et al., 2018).

Gene synteny and gene structure results showed that conserved gene clusters, i.e., *cul4a*-*lamp1*-*grtp1*-*adprhl1*-*dcun1d2*-*tmco3*, *clgaltlc1*-*mcts1*-*cul4b*-*lamp2*-*atp1b4*-*tmem255a*-*zbt33*, *lamp3*-*mccc1*-*dcun1d1*-*atp11b*-*sox2*, which belonging to *lamp1*-*3* (Figures 6A–C), respectively, were observed in almost representative vertebrate genomes, implying that the *lamp1*-*3* genes may be exhibited a highly conserved synteny during evolution. However, some flanking genes of the *M. salmoides lamp1*-*3* were arranged differently or lost compared with the other fishes or vertebrates (Figure 6), which might be the result of the gene deletion or gene rearrangement event (Hu et al., 2022), and showing that the largemouth bass has an independent evolutionary history.

Exploring the mRNA distribution patterns of *lamp1-3* genes in different tissues would be beneficial to understand the role of these genes in largemouth bass. The tissue expression patterns of the three genes were analyzed by qRT-PCR. The results showed that the *lamp1* gene was widely distributed in all tissues examined, and the highly transcript level of *lamp1* was found in the brain (Figure 7A), which is in line with previous studies in Japanese flounder (Rondón-Barragán et al., 2017), indicating that the *lamp1* gene may be involved in neural activity. For instance, Barrachina et al. (2006) revealed that the transcript level of *lamp1* was increased in neurons and in glial cells surrounding senile plaques in patients with Alzheimer’s diseases. Furthermore, the high expression of *lamp1* mRNA in the muscle suggested that it may also be involved in myogenesis, which was consistent with a study carried out by Sakane and Akasaki (2018), who demonstrated that knocked down of *lamp1* decreased the expression levels of myogenic regulatory factors in C2C12 myotube formation. However, the function of *lamp1* in other tissues and its expression pattern in fish is still unknown.

In vertebrates, *lamp2* mRNA expression has been documented in several tissues/organs, such as neural crest derived ganglia, liver, pancreas and kidney in murine (Lichter-Konecki et al., 1999), heart and brain in bird (Hatem et al., 1995), and C2C12 myoblasts in mouse (Sakane and Akasaki, 2018). Similarly, *lamp2* transcription was extensively expressed in all selected tissues in largemouth bass, and abundantly exhibited in heart, muscle and spleen, indicating that it might may play a critical role in these tissues. In human, accumulating evidences revealed that *lamp2* deficiency induced hypertrophic cardiomyopathy and eventually led to the Danon disease (Zhai et al., 2023). In addition, previous studies showed that *lamp2* was involved in the differentiation process of C2C12 myoblasts to myotube (Sakane and Akasaki, 2018). Whether the *lamp2* has the same function in teleosts requires further investigation.

In contrast to the ubiquitous *lamp1* and *lamp2*, *lamp3* mRNA expression has been reported to be tissue and species specific. For instance, in mammals, previous studies have observed that higher expression of *lamp3* in various cancer tissues (carcinomas) (Nagelkerke et al., 2011; Wang et al., 2017; Gui et al., 2018; Nakamura et al., 2023a). Salaun et al. (2003) have shown that mouse *lamp3* mRNA is expressed almost exclusively in peripheral cells of the lung, but is not detected in other tissues. de Saint-Vis et al. (1998) have found that *lamp3* expression levels are induced upon activation of human dendritic cells. Other studies have indicated that the chicken *lamp3* is expressed in a wide range of tissues, including lung, thymus, spleen, bursa and ceacal tonsil (Wu et al., 2010). In the present study, we observed that Ms-*lamp3* was constitutively distributed in all tissues examined, with the highest expression in spleen, and higher in liver and kidney tissues, which is consistent with the study by Johansson et al. (2012), which revealed that the *lamp3* transcription was highest in spleen, kidney and liver of *O. mykiss*, and was induced by infection with viral and bacterial pathogens, referring that *lamp3* may play a critical immune role in largemouth bass. In addition, the *lamp3* was highly expressed in the heart, we speculated that it also has a pivotal role in this tissue, but its need further study.

Many studies have been clearly elaborated that the starvation or food-deprived often influenced the function of immunity in mammals (Han et al., 2021; Loft et al., 2022; Forslund, 2023; Janssen et al., 2023) and altered their energy metabolism (Browning et al., 2012; Soeters et al., 2012; Dote-Montero et al., 2022). Similarly, the aforementioned phenomena have also been observed in teleosts (Dai et al., 2018; Liao et al., 2021; Wang et al., 2021; Gou et al., 2023; Shi et al., 2023). Moreover, although some studies have shown that starvation alters the expression of the autophagy-related genes in fish (Balmori-Cedeño et al., 2019; Wu et al., 2020; Pan et al., 2022), there are still many autophagy-related gene from teleosts that have not been identified and characterized. In our study, we observed that the expression of *lamp1* in the liver was significantly induced after starvation, and then returned to normal levels after refeeding (Figure 8A). This finding was consistent with previous studies in zebrafish (Fan et al., 2020), suggesting that the *lamp1* gene may be involved in energy regulation via autophagy in largemouth bass. To validate that autophagy occurred after long-term starvation, the transcription level of two classical autophagosomal markers, *Beclin1* and *MAPLC3* (Yabu et al., 2012; Al-Shenawy, 2016), was detected in three tissues (liver, spleen and kidney) of largemouth bass in the present study. As expected, the mRNA levels of both of autophagosomal marker genes were significantly increased in the three tissues after the fasting treatment, and then remarkably decreased after the refeeding treatment (Figure 9). Interestingly, the *lamp2* and *lamp3* showed the same expression pattern trends as *lamp1* in the liver tissue (Figures 8D, G). The liver is a multi-functional organ with roles in metabolism, nutrient storage, innate immunity and detoxification in vertebrates (Robinson et al., 2016; Causey et al., 2018). The high expression levels of *lamp* genes in the liver were induced by long-term food deprivation, which might facilitate the fish to maintain energy homeostasis during starvation. Collectively, we speculated that *lamp1-3* genes might be involved in energy regulation in the liver via the autophagy pathway in largemouth bass and these results provide a novel insight into the mechanism of the adaptive starvation in this fish species.

To investigate whether or not the *lamp1-3* genes are involved in the immune progression in largemouth bass, we determined their transcriptional patterns in response to starvation and refeeding schedules. In the present study, the expression of *lamp1* in the largemouth bass kidney was remarkably decreased after starvation and refeeding compared to the control group. On the contrary, the transcript level of *lamp2* and *lamp3* in kidney was significantly increased after a long-term food deprivation, and then strikingly decreased to the same level or even lower than that of the control group after refeeding (Figures 8F, I), suggesting that the *lamp2* and *lamp3* but not the *lamp1*, play a priority role in the kidney tissues under the stress condition (long-term food deprivation) via autophagy. In addition, we found that there was no significant difference in the mRNA expression levels of both *lamp1* and *lamp3* in the spleen of largemouth bass between the control and fasted group (Figures 8B, H), but the transcript level of *lamp2* was remarkably increased in the fasted treatment group and slightly decreased in the refed treatment group (Figure 8E), indicating that the *lamp2* might play an important role in the spleen. The above expression patterns were similar to previous findings of *IL-8* in kidney and *IL-6 and IL-4* in the spleen piglets after dietary restriction treatment (Han et al., 2018). Kidney is an important haematopoietic tissue with the ability to sample and retain antigens (Bjørgen and Koppang, 2021) and plays a vital role in immunity. The spleen is considered to be a primordial secondary lymphoid organ, generating adaptive immune responses in almost all gnathostomes (Flajnik, 2018). Previous studies have been demonstrated that the autophagy-related genes were also induced in the kidney and spleen when fish were challenged with viruses (Kong et al., 2011; Zhang et al., 2022), bacteria (Qin et al., 2016) or stress conditions (Kumar et al., 2022; Kumar et al., 2023). It is therefore reasonable to speculating that the *lamp2* and *lamp3* genes may also have an important immunity function in largemouth bass, which needs to be further investigated.

## 5 Conclusion

In summary, the *lamp1-3* gene family of largemouth bass (*M. salmoides*) has been identified and characterized. Multiple alignment of protein sequences, 3-D structural model prediction, gene synteny and phylogeny were performed, showing that the *lamps* genes are conserved across vertebrates. In addition, the distribution of these genes was analyzed by q-PCR, indicating that three genes are extensively detected but showed different patterns. Furthermore, the fasting and refeeding schedule experiments demonstrated that three *lamp* genes may have different functions in immune and energetic organisms in largemouth bass.

## Data Availability

The datasets presented in this study can be found in online repositories. The names of the repository/repositories and accession number(s) can be found in the article/Supplementary Material.
