# Supplementary material for: Characterization of three lamp genes from largemouth bass (Micropterus salmoides): molecular cloning, expression patterns, and their transcriptional levels in response to fast and refeeding strategy
Source: Front Physiol. 2024 Apr 5;15:1386413. doi: 10.3389/fphys.2024.1386413 (PMC11026864; doi:10.3389/fphys.2024.1386413)
Supplement: Supplementary file 1 [file DataSheet1.pdf]

Supplementary Table. 1 NCBI and Ensemble accession numbers of protein sequences.

Species	Protein	accession numbers
Homo sapiens	LAMP1	AAH93044.1
Homo sapiens	LAMP2	AAB41647.1
Homo sapiens	LAMP3	AAH32940.1
Pan troglodytes	LAMP1	*JAA37074.1*
Pan troglodytes	LAMP2	JAA43094.1
Pan troglodytes	LAMP3	NP_001315221.1
Bos taurus	LAMP1	AAI33620.1
Bos taurus	LAMP2	AAI02820.1
Bos taurus	LAMP3	NP_001095605.1
Rattus norvegicus	LAMP1	BBJ00845.1
Rattus norvegicus	LAMP2	AAH61990.1
Rattus norvegicus	LAMP3	AAH83787.1
Aythya fuligula	LAMP1	XP_032057667.1
Aythya fuligula	LAMP3	XP_032049240.1
Meleagris gallopavo	LAMP1	XP_003203252.1
Meleagris gallopavo	LAMP2	XP_031410597.1
Meleagris gallopavo	LAMP3	XP_010715159.1
Columba livia	LAMP1	XP_021147261.1
Columba livia	LAMP2	PKK26942.1

(Continue)

Species	Protein	accession numbers
Columba livia	LAMP3	PKK27053.1
Sceloporus undulatus	LAMP1	XP_042312276.1
Sceloporus undulatus	LAMP2	XP_042334331.1
Sceloporus undulatus	LAMP3	XP_042313018.1
Lacerta agilis	LAMP1	XP_033003617.1
Lacerta agilis	LAMP2	XP_032993207.1
Lacerta agilis	LAMP3	XP_033006628.1
Carassius auratus	LAMP1	XP_026111278.1
Cyprinus carpio	LAMP1	XP_018967281.1
Cyprinus carpio	LAMP2	XP_042626170.1
Cyprinus carpio	LAMP3	XP_018920582.1
Ctenopharyngodon idella	LAMP1	QJD55563.1
Ctenopharyngodon idella	LAMP2	QJD55565.1
Danio rerio	LAMP1	NP_955996.1
Danio rerio	LAMP2	AAH90897.1
Danio rerio	LAMP3	XP_021329231.1
Salvelinus alpinus	LAMP1	XP_023866460.1
Salvelinus alpinus	LAMP2	XP_023846014.1
Salvelinus alpinus	LAMP3	XP_023860089.1

(Continue)

Species	Protein	accession numbers
Salmo salar	LAMP1	NP_001158846.1
Salmo salar	LAMP2	XP_014067225.1
Salmo salar	LAMP3	XP_014072251.1
Oncorhynchus mykiss	LAMP1	XP_036837844.1
Oncorhynchus mykiss	LAMP2	XP_021439878.2
Oncorhynchus mykiss	LAMP3	XP_021454939.2
Oryzias latipes	LAMP1	XP_004067118.1
Oryzias latipes	LAMP2	XP_023814775.1
Oryzias latipes	LAMP3	XP_011472488.1
Larimichthys crocea	LAMP1	TMS12843.1
Larimichthys crocea	LAMP3	XP_010734916.3
Perca flavescens	LAMP1	XP_028427632.1
Perca flavescens	LAMP2	XP_028444969.1
Perca flavescens	LAMP3	XP_028443583.1
Rana temporaria	LAMP1	XP_040192186.1
Rana temporaria	LAMP2	XP_040179879.1
Xenopus tropicalis	LAMP1	NP_001135713.1
Xenopus tropicalis	LAMP2	XP_041430205.1
Xenopus tropicalis	LAMP3	XP_018117942.2

(Continue)

Species	Protein	accession numbers
Gallus gallus	LAMP2	NP_001384687.1
Siniperca chuatsi	LAMP2	XP_044060848.1
Siniperca chuatsi	LAMP3	XP_044056292.1
Morone saxatilis	LAMP2	XP_035525454.1
Morone saxatilis	LAMP3	XP_035509772.1
Sparus aurata	LAMP2	XP_030253165.1
Oryzias melastigma	LAMP1	KAF6725489.1
Oryzias melastigma	LAMP2	KAF6732397.1
Oryzias melastigma	LAMP3	KAF6724323.1
Lampetra japonicum	LAMP1	*ENSPMAG00000001233*

Supplementary Table 2. Amino acid sequence identifies comparison of largemouth bass LAMP1 putative peptides with other vertebrates. The top right refers to the amino acid identify, and the bottom left represents the amino acid divergene.

	1	2	3	4	5	6	7	8	9	10	11	12	13	14	15	16	17	18
1.*M_salmoides*		43.5	40.7	41.5	44.9	43.4	43.3	54.3	69.1	69.3	82.5	88.9	85.8	68.8	88	43.2	83.2	99
2.*H_sapiens*	99		43.2	66.8	50.7	49.9	72.9	42.9	41.2	41.7	43.3	43.3	44.5	42.3	43.8	64	44.8	43.5
3.*R_temporaria*	108.7	100.1		41.2	44.6	46	43.6	39.4	39.8	40	40.5	39.9	40.7	41.7	41	44.5	41	40.5
4.*R_ norvegicus*	105.8	43.6	107.1		44.4	46.1	65.2	42.1	40	39.8	40.3	40.8	41.5	41.7	41.5	56.7	41.1	41.5
5.*C_livia*	94.7	77.9	95.6	96		56.7	47.4	42.7	40.7	41	42.5	43.8	42.4	41.5	44.1	49.1	43.7	44.6
6.*S_undulatus*	99.5	80.2	91.1	90.9	63.5		48.5	41.5	43.1	43.6	42.9	42.8	42.4	41.7	43.9	50.4	42.9	43.1
7.*B_taurus*	99.7	33.7	98.9	46.6	87	84		43.8	42	42.5	42.9	42.3	44.1	42.8	43.8	59.5	44.6	43.3
8.*D_rerio*	69	101.1	113.8	103.7	101.7	105.8	98		53.2	54.3	52.6	53.3	53.3	49	54.6	42.6	52.5	54.6
9.*S_salar*	39.8	107	112.2	111.3	108.7	100.3	104.3	71.7		95.2	67.3	67.7	66.2	58.6	68.4	41.4	66.3	69.1
10.*O_mykiss*	39.4	105.2	111.3	112.2	107.9	98.6	102.6	69.1	4.9		68.5	68.7	66.4	58.8	68.6	41.2	66.3	69.3
11.*L_crocea*	20	99.7	109.6	110.3	102.6	101	101.3	73.2	42.8	40.7		81	78.8	67.7	81.8	43.3	77.1	81.8
12.*S_aurata*	12	99.9	111.8	108.5	98.1	101.5	103.1	71.3	42.1	40.5	21.9		83.9	68.3	85.3	42.3	80.3	88.5
13.*P_flavescens*	15.8	95.9	108.7	105.8	102.7	102.8	97.3	71.3	44.8	44.4	24.9	18.2		68.8	87.5	44	81.5	85.6
14.*O_niloticus*	40.3	103.1	105.2	105.2	106	105.4	101.6	82.6	59.5	59	42.2	41.1	40.3		70	42.8	69.4	69
15.*E_lanceolatus*	13.1	98.2	107.9	105.8	97	97.8	98.1	68.4	41	40.6	21	16.4	13.7	38.3		44	82	87.5
16.*O_anatinus*	100	48.8	96	63.7	82.2	78.9	57.7	102.1	106.3	107.1	99.8	103.3	97.5	101.5	97.5		44	43.2
17.*C_argus*	19.1	94.9	107.8	107.6	98.4	101	95.5	73.5	44.5	44.5	27.4	22.9	21.3	39.3	20.6	97.4		
18.*M_dolomieu*	1	99	109.6	105.8	95.4	100.3	99.7	68.4	39.8	39.4	21	12.6	16.1	39.9	13.7	100	18.7	

Supplementary Table 3. Amino acid sequence identifies comparison of largemouth bass LAMP2 putative peptides with other vertebrates. The top right refers to the amino acid identify, and the bottom left represents the amino acid divergene.

	1	2	3	4	5	6	7	8	9	10	11	12	13	14	15
1.*M_salmoides*		39.4	40.9	36.5	44.4	38.3	41.2	40.7	56.6	56.6	59.2	71.2	69.5	76	41.8
2.*H_sapiens*	113.6		42.1	67.5	47.2	44.9	76.4	32	37.6	36.9	36.9	38.9	39.6	40.4	31.1
3.*R_temporaria*	108.3	103.8		38.6	48.1	40.5	45.5	35.8	34.2	33.7	36.5	36.6	38	36.5	34.9
4.*R_ norvegicus*	125.5	42.5	117.1		43.8	40.6	62.8	30.5	38.1	37.1	37.3	37.5	36.5	38.5	29.7
5.*C_livia*	96.1	87.5	85.1	98		48.8	50.1	33.1	38.9	40	39.3	38.1	41.6	41.3	31.9
6.*S_undulatus*	118	94.5	109.4	109.1	83.1		42.6	31.1	37.1	37.1	37	38.7	39	38.3	30.6
7.*B_taurus*	106.9	28.3	92.6	51.1	79.5	102.1		35.6	35.8	36	36.3	35.2	35.8	36.1	34.1
8.*D_rerio*	108.9	148.1	128.8	156.5	141.9	153.1	130		32.7	32.4	33.4	35.7	33.7	34.5	71.3
9.*S_salar*	63.8	120.9	136.6	119.1	115.8	123	129.1	144.4		93.5	81.7	61.4	58.9	61.1	33.2
10.*O_mykiss*	63.8	124.1	139.2	123.3	111.5	123	128	146	6.8		79.5	60.9	58.7	60.6	32.7
11.*S_alpinus*	58.2	124.1	125.6	122.4	114	123.5	126.6	140.4	21	24		60.2	60.2	60.2	35.4
12.*P_flavescens*	36.2	115.8	125.1	121.4	119	116.6	131.5	129.5	53.7	54.7	56.1		71	80.5	37.7
13.*S_aurata*	39.2	112.8	119.2	125.6	105.8	115.2	128.9	138.9	58.8	59.3	56.1	36.6		75.1	35.7
14.*S_chuatsi*	28.9	110	125.6	117.2	106.8	118.2	127.7	135.1	54.3	55.3	56.1	22.6	30.3		36.2
15.*C_idella*	105	153	132.9	161.8	148.5	156.4	137	36.2	141.4	144.4	130.6	120.7	129.3	127.2	

Supplementary Table 4. Amino acid sequence identifies comparison of largemouth bass LAMP3 putative peptides with other vertebrates. The top right refers to the amino acid identify, and the bottom left represents the amino acid divergene.

	1	2	3	4	5	6	7	8	9	10	11	12	13	14	15
1.*M_salmoides*		25.7	28.4	26	27.9	26.7	42.1	46.6	47.8	70	73.9	73.2	65.6	87.5	24.4
2.*H_sapiens*	191.9		54.9	25.4	27.1	63.2	26	28.5	28.5	26.4	27.2	25.8	25.2	27	34.2
3.*R_norvegicus*	170.7	67.7		24.2	26.6	48.6	24.2	27.2	28	27.9	28.7	30.6	25.4	30.2	28.5
4.*C_livia*	189.3	195	202		34.3	27.9	24.3	27.6	28.4	28.4	27.9	26.1	24.7	28.2	40.4
5.*P_ vitticeps*	174.5	180	184.6	136.1		27.8	29.7	27.1	27.1	28.2	26.9	27.6	26.1	28.8	31.5
6.*B_taurus*	183.4	50.3	83.5	174.3	175.1		29	28.3	27.9	27.9	29	28.5	27	28.5	38.6
7.*D_rerio*	104	189.4	202	200	161.7	166.7		39.8	41.7	43.4	42.7	40.8	42.5	41	27.2
8.*S_salar*	89.4	169.9	179.8	176.3	180.7	171.4	112.4		96.2	44.8	44.5	43.6	44.3	46	25.7
9.*O_ mykiss*	85.9	169.9	173.9	170.6	180.7	174.2	105.1	3.9		46.4	46.9	44.8	44.3	47.2	26.1
10.*L_ crocea*	38.3	185.6	174.2	170.7	172	174.5	99.5	95.1	90.1		70	61.4	61.3	72.3	26.8
11.*S_aurata*	32.1	179.6	168.6	174.2	182	166.2	101.7	95.9	88.4	38.2		66	62.2	75.5	28
12.*P_flavescens*	33.1	191	156.4	188.3	176.7	170.3	108.5	98.8	94.9	53.8	45.1		59.8	77.5	25.3
13.*O_niloticus*	45.8	196.6	194.7	197	188.6	181.5	102.6	96.6	96.6	54	52.1	56.9		66.3	25.5
14.*S_chuatsi*	13.8	180.8	158.9	172.4	167.8	170.3	107.6	91.2	87.6	34.5	29.7	26.8	44.6		25.3
15.*O_anatinus*	200	136.6	170	110	151.1	117	179.9	192	188.5	182.7	173.4	195.5	193.7	195.5	
